# Navigating Health Care with Concurrent Jaw and Neck Pain: A Patient-Centered Perspective

**DOI:** 10.1177/23800844251365594

**Published:** 2025-09-15

**Authors:** A. Böthun, A. Fjellman-Wiklund, J. Durham, F. Hellström, B. Häggman-Henrikson, A. Lövgren

**Affiliations:** 1 Department of Odontology, Orofacial Pain and Jaw Function, Faculty of Medicine, Umeå University, Umeå, Sweden; 2 Department of Community Medicine and Rehabilitation, Physiotherapy, Umeå University, Umeå, Sweden; 3 School of Dental Sciences, Newcastle University, Newcastle, UK; 4 Newcastle Hospitals NHS Foundation Trust, Newcastle, UK; 5 Department of Community Medicine and Rehabilitation, Umeå University, Umeå, Sweden; 6 Department of Occupational Health, Psychology and Sports Sciences, Faculty of Health and Occupational Studies, University of Gävle, Gävle, Sweden; 7 Department of Orofacial Pain and Jaw Function, Faculty of Odontology, Malmö University, Malmö, Sweden

**Keywords:** chronic pain, facial pain, neck pain, pain management, qualitative research, temporomandibular joint disorders

## Abstract

**Introduction:**

Concurrent pain in the jaw and neck is common; however, little is known about how it develops. Previous studies have focused mainly on biological factors with limited focus on psychosocial factors and patients’ perspectives. To enhance understanding of concurrent jaw and neck pain, knowledge is needed about patients’ perspectives on the development of symptoms and their management within health care that also includes dentistry.

**Objective:**

To explore patients’ perspectives on the development of concurrent jaw and neck pain in relation to navigating the health care system.

**Methods:**

Sixteen individuals (11 women and 5 men, aged 19 to 56 y) with concurrent jaw and neck pain were recruited using purposive sampling. Participants were patients referred to an orofacial pain specialist in Sweden. Individual semi-structured interviews were used and analyzed using qualitative content analysis.

**Results:**

Data analysis resulted in the theme “Seeking understanding, confirmation, and simplified navigation in the health care system.” The main theme consisted of 3 subthemes: (1) understanding and interpreting the body and emotions holistically, (2) seeking affirmation and legitimacy from health care, and (3) wishing for clear and easily navigable health care pathways. Patients expressed thoughts regarding their own sensemaking in respect to causes for their pain. Moreover, they requested confirmation from health care along with simplified health care navigation and a holistic approach regarding pain management.

**Conclusions:**

A mismatch exists between the patient’s own sensemaking regarding the cause of their jaw and neck pain and the care they receive from their health care provider. Having pain from multiple sites that is managed in different medical systems makes it difficult to navigate within health care. A clear care pathway could reduce the risk of pain chronicity, and an increased collaboration between dentistry and medical care would be desirable.

**Knowledge Transfer Statement:**

Concurrent jaw and neck pain is common but often managed separately in dentistry and health care. This can result in a mismatch between a patient’s understanding regarding concurrent jaw and neck pain and directions from their health care provider. A holistic approach and increased collaboration between dentistry and health care are needed to support patients navigating health care providers and reduce the risk of pain chronification.

## Introduction

Pain is one of the main reasons why patients seek health care (Wiitavaara et al., 2017). In dentistry, the most frequently managed acute orofacial pain condition is toothache, whereas the most common chronic orofacial pain condition is temporomandibular disorder (TMD). As with other chronic pain conditions, TMD is associated with psychosocial comorbidities (Slade et al. 2020) along with overlap in pain locations where pain in the neck is one of the most frequent pain sites in relation to TMD (Lövgren et al., 2022). The connection between the jaw and neck (Miçooǧulları et al. 2024a; Miçooǧulları et al. 2024b) is also reflected by the strong correlation between the jaw and neck in pain severity (Visscher et al. 2001). In addition, patients with concurrent jaw and neck pain report higher levels of psychological distress than do patients with localized TMD pain (Visscher et al. 2001). However, the mechanisms behind pain spreading and development are not fully understood, but the close anatomical relation between the regions or a consequence of central sensitization have been suggested (Slade et al. 2020). In this regard, previous studies have focused primarily on the biological aspects for pain onset such as trauma, genes, and hormones. More recent studies, however, have suggested that psychosocial factors such as the level of depression and stressful life events may significantly contribute to pain development (Tanguay-Sabourin et al. 2023). Currently, it is virtually unknown which factors patients perceive as important in relation to pain development and spreading. In relation to the first onset of pain, early identification and intervention have been suggested as key steps in improving the long-term outcomes among patients. Although such patient-centered care is encouraged, patients with TMD report difficulties receiving a diagnosis (Durham et al. 2010). This in turn may cause increased worry about their condition—a worry that alone may contribute to increased pain. Collectively, this highlights the need for an early and potentially multidisciplinary approach incorporating interventions such as information and self-management (Beecroft et al. 2024).

In health care, jaw and neck pain, although commonly related, are mostly managed as 2 separate constructs. From a dental care perspective, diagnosing TMD that presents as a single condition is reportedly challenging due to the complexity of the symptoms (Ilgunas et al. 2021). From a health care perspective, neck pain is also considered challenging, and oral health including jaw pain is currently not included in standard routines and guidelines (Wu et al. 2024). However, in pain assessment and management, especially due to the subjective nature of pain, a holistic approach that takes into account both mental and social factors as well as the purely physical ones is a necessity. Therefore, knowledge of the patient’s perspective of receiving care for different pain complaints is relevant to improve future management.

Having pain that is managed in 2 different organizational health care systems, such as dentistry and health care, could further complicate health care navigation. One such example is that patients with TMD pain as a single symptom express that they struggle in finding the right health care provider for their complaints (Durham et al. 2011; Rollman et al. 2013). In dentistry, individuals with TMD often go undetected and undertreated. In addition, patients with persistent TMD express that they receive inconsistent recommendations when seeking care (Penlington et al. 2024). Uncertainty while seeking health care is also mentioned among individuals with lower back pain, which highlights the importance of the interaction between the health care provider and the patient (Costa et al. 2023). Moreover, it is necessary to gain insight regarding which patient-reported outcomes the patients perceive as most important in symptom development and in their health care process (Häggman-Henrikson et al. 2022; Hickey et al. 2023). Although concurrent jaw and neck pain is common, most studies focus on trauma-related neck pain. However, studies on concurrent jaw and neck pain, regardless of cause, are rare. Using a qualitative study design, a deeper understanding of elements of importance to patients could be gained, which in turn may provide new insights for the development of future interventions (Dahlgren et al. 2019). Therefore, the aim of the present study was to explore patients’ perspectives on the development of concurrent jaw and neck pain in relation to navigating the health care system.

## Methods

### Ethics

The study was approved by the Swedish Ethical Review Authority (Dnr 2023-05095-01) and was conducted in accordance with the World Medical Association Declaration of Helsinki. Oral and written informed consents were obtained from participants prior to participation, and the participants had the right to remove data if requested. The study followed the Standards for Reporting Qualitative Research. (SRQR)

### Recruitment

The participants were patients recruited from the specialist and student clinics at the Department of Clinical Oral Physiology, Umeå University Hospital, Sweden. All patients at these clinics are referred from primary or secondary care, either from a dentist or a medical doctor. Between August 7, 2023, and November 24, 2023, 155 patients (122 women and 33 men, 12 to 83 y) underwent an intake examination according to the Diagnostic Criteria for Temporomandibular Disorders (DC/TMD) (Schiffman et al., 2014) at their first visit at either the specialist clinic or student clinic. The inclusion criteria were (1) age 18 to 70 y, (2) able to understand spoken and written Swedish language, (3) reporting of concurrent jaw and neck pain according to a pain drawing on a body mannequin, and (4) reporting familiar pain on palpation over the jaw and neck or familiar pain during jaw movements during the clinical examination (Schiffman et al., 2014). To maintain a lay perspective among the participants, exclusion criteria included health care personnel or researchers working within the pain field. Most of the participants were excluded due to not reporting familiar jaw or neck pain. Individuals were also excluded if they had been diagnosed with arthralgia or arthritis in the temporomandibular joint (Schiffman et al., 2014) or had a diagnosed rheumatic disease, which resulted in our cohort being predominantly, but not exclusively, myogenous.

**Figure 1.**
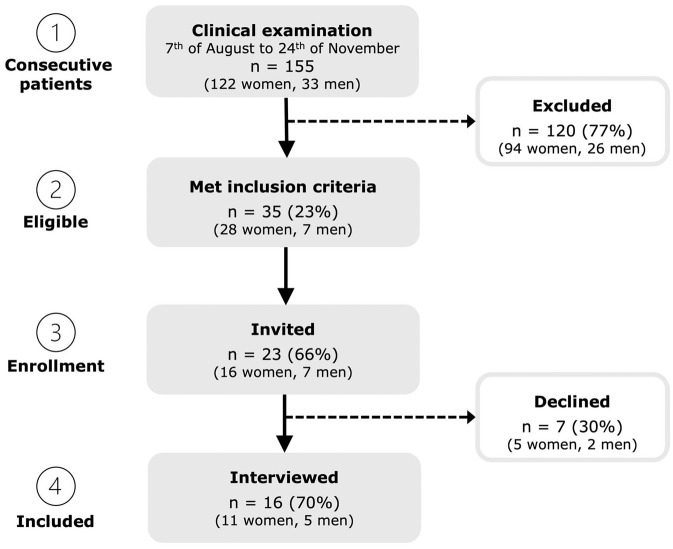
Flowchart illustrating the recruitment process (consecutive, eligible, invited, and interviewed participants).

Posters advertised the study in public areas at the specialist and student clinics. Thirty-five individuals met the inclusion criteria during the recruitment period, and of these, 23 were contacted via telephone by the first author (A.B.) to inquire about their willingness to participate in the study (Fig. 1). A purposive sampling was used that included maximum variation in gender, age, and education level among the participants to obtain a depth and breadth of views and experiences among the interviewed individuals (Dahlgren et al. 2019). Participants were not required to give a reason for declining participation. Sixteen individuals accepted and were interviewed between December 2023 and January 2024. Participants reported a pain duration of 3 mo to 20 y. Pain duration also varied within age; for example, among participants 40 y and older, the pain duration varied between 1 y and 20 y. Characteristics of the study population including gender, age, education level, and diagnosis according to the DC/TMD are presented in Table 1.

**Table 1.** Characteristics of Study Population.

	Women (*n* = 11)	Men (*n* = 5)	Total (*n* = 16)
Age (y)	19–56	22–56	19–56
Education level			
Elementary school or secondary school	8	4	12
University degree	3	1	4
DC/TMD diagnosis			
Myalgia	11	5	16
Headache attributed to TMD	5	3	8
Disc displacement with reduction	2	2	4
Disc displacement without reduction	1	0	1

DC/TMD, Diagnostic Criteria for Temporomandibular Disorders.

### Data Collection

Data were collected using individual semi-structured interviews (Kvale and Brinkmann 2009). A semi-structured thematic guide was used (Dahlgren et al. 2019), and the participants were allowed to share their experiences in relation to several questions organized in different themes regarding concurrent jaw and neck pain that included cause, development, daily life, treatment, and management. The thematic guide had been designed and discussed among the researchers and was later tested during 2 pilot interviews and revised before data collection. Before participation, the participants were informed about the aim of the study and that they had been invited due to reporting concurrent jaw and neck pain at their clinical examination. The participants were also assured anonymity during analysis and in the presentation of the results. They were also informed about the opportunity of reading a summary of the interview afterwards, which one participant requested. Participants were not given the opportunity to provide feedback on the findings of the study but were informed that the results would be part of future scientific publications.

All interviews were performed by the first author (A.B.), who was not responsible for or involved in the care of the participants. The interviews were performed in a manner most convenient for the participant (i.e., face-to-face in a conference room at the Department of Odontology, Umeå University, via telephone or using online video conferencing). All interviews were performed individually between the first author and the participant except for 1 interview in which the participant requested an accompanying friend to be present; the accompanying friend remained quiet during the interview.

All interviews were performed on 1 occasion, except for 1 interview. One follow-up interview was performed with 1 participant due to issues with the audio recording; this follow-up interview was conducted on the same day. With the participants’ consent, the interviews were recorded, and the participants were assigned with a code to preserve anonymity. Open-ended follow-up questions were used to help participants to further explore their experiences. The interviews lasted from 23 to 70 min. The interviews were transcribed verbatim by a transcriber in close connection to the interviews, and all transcribed interviews were later checked for accuracy by the first author. The interviews were performed and initially analyzed in the Swedish language. The transcriptions were not translated to English. The analysis and quotes were later translated to English by a professional and translated back to Swedish including a back-translation check.

### Data Analysis

The interviews were analyzed using qualitative content analysis (QCA), a systematic method of analyzing written or verbal communication that is suitable for studying experiences and opinions (Graneheim et al., 2004; Graneheim et al., 2017). QCA focuses on differences between and similarities within codes and categories. The method allows both manifest and latent interpretations of content, but the interpretations vary in depth and level of abstraction (Graneheim et al., 2004; Graneheim et al., 2017).

At first, all transcribed interviews were read independently by four researchers (A.B., A.F.W., A.L., F.H.) several times to achieve an overall idea of the entity. The transcribed data relevant to the research question was divided into meaning units that consist of several words or sentences regarding a specific content (Table 2). In the next stage, the meaning units were condensed and coded independently by the 4 researchers. Coding is the stage at which the condensed meaning units are labeled (Table 2). Codes were interpreted and compared for differences and similarities and sorted into categories and subthemes depending on the meaning. The codes, categories, and subthemes were discussed among all authors using an iterative approach. Finally, an abstraction was performed to formulate a latent theme at a higher interpretation level, which was discussed by the researchers until a negotiated outcome was agreed upon. Triangulation between the researchers was used to minimize subjective interpretations and the influence of diverse backgrounds and expertise of the researchers (Lincoln and Guba 1985). Researchers involved in the analysis had different backgrounds and professions as follows: 2 were dentists with knowledge in research and management of patients with orofacial pain (1 was a general dentist [A.B.] and the other was a dentist who specialized in orofacial pain and had experience in qualitative research [A.L.]); 1 was a physiotherapist with experience in qualitative research, work-related pain, and management of pain patients (A.F.W.); and 1 was a researcher with knowledge of work-related pain and neck pain in particular (F.H.). As part of the triangulation process, the manuscript was also read by the transcriber to check the accuracy of the findings in relation to the original transcriptions.

**Table 2.** Example of Condensed Meaning Unit, Codes, Category, and Subtheme.

Condensed Meaning Unit	Codes	Category	Subtheme
I had started a new job . . . working in a completely new place . . . new people, new tasks, it was the first job I had that was . . . connected to my education . . . felt like I had a lot to prove	Challenges associated with: New job New place New people New tasks First job Lots to prove in new job	Association between pain onset and life event	Understanding and interpreting the body and emotions holistically

## Results

**Figure 2.**
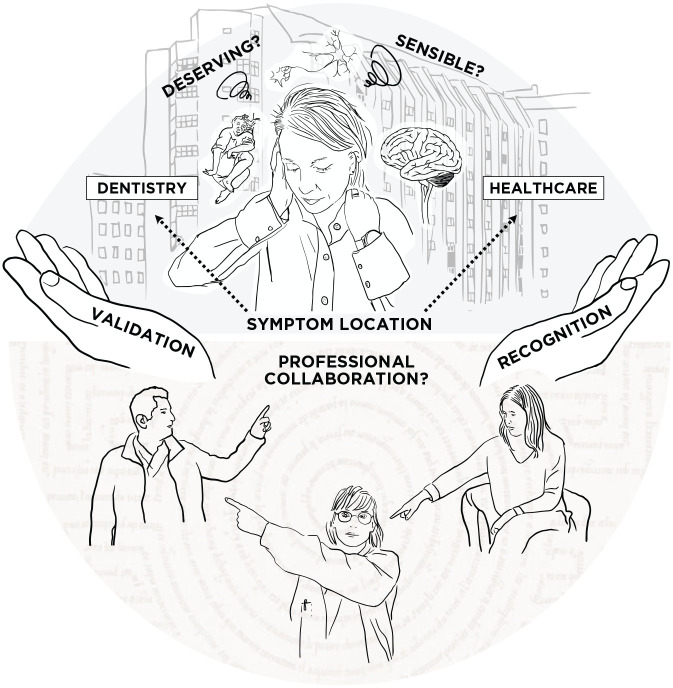
The main theme, “Seeking understanding, confirmation, and simplified navigation in the health care system,” is exemplified showing the patient’s own sensemaking (upper part), the patient’s wishes from health care in terms of validation and recognition, and the patient’s thoughts regarding health care navigation (lower part).

Our analysis resulted in a main theme, “Seeking understanding, confirmation, and simplified navigation in the health care system,” which was based on 3 subthemes (“Understanding and interpreting the body and emotions holistically,” “Seeking affirmation and legitimacy from health care,” and “Wishing for clear and easily navigable health care pathways”), and 8 categories (Table 3). The main theme entails the patient’s own sensemaking regarding the cause and development of their symptoms, asking for validation and recognition from health care, and a need for simplified health care navigation (Fig. 2).

**Table 3.** Table Illustrating the Theme, 3 Subthemes, and 8 Categories.

Seeking Understanding, Confirmation, and Simplified Navigation in the Health Care System							
Understanding and Interpreting the Body and Emotions Holistically			Seeking Affirmation and Legitimacy from Health Care			Wishing for Clear and Easily Navigable Health Care Pathways	
Association between pain onset and life event	Personal understanding of cause	Body and emotions—closely connected	Desire to be seen and understood	Need for explanations	Strategies for getting help when seeking care	Care seeking based on symptoms	Unclear and suboptimal collaboration

### Understanding and Interpreting the Body and Emotions Holistically

The participants expressed that they were trying to understand the cause of their jaw and neck pain and remembered a certain time point for pain onset or symptom exacerbation. In their own sensemaking, their symptoms were often connected to a certain life event that resulted in increased stress or a time in their life when many things were happening at the same time. Life events mentioned by the participants were starting a new job, a death in the family, or an injury.

The trauma of grieving for my father while having a new job in another country, so there was a lot of stress. (Participant 3)

The participants expressed their thoughts and ideas regarding the cause and start of pain. For some, the jaw pain started with toothache, for example, due to a cracked tooth, whereas for others, it started with neck pain after a physiotherapist mobilized the neck. Others had problems identifying the cause of their pain since the pain could not be related to a specific event. The participants reflected on possible contributing factors such as ergonomics at work, general bodily tension, iron deficiency, hormonal imbalance, or general stress.

Stress . . ., fatigue, bad luck, hard to say. As I said, I haven’t been involved in anything, I haven’t been hit, I haven’t fallen, it’s not like anything has happened, like trauma to the jaw, really. (Participant 9)

Participants expressed a belief that their jaw and neck pain were connected. To them, this interconnectedness was not solely related to the physical symptoms but also was on a holistic level (i.e., including mind and body). The participants thought that symptoms were connected to their overall mental health, for example, experiencing more intense pain during a depression or exhaustion syndrome and experiencing less pain when feeling better mentally.

If I am depressed, I am convinced that my symptoms are getting worse. That my back hurts more, my neck hurts more and so on, that it becomes more intense. But I don’t think that . . . the pain goes away just because my mood improves. But rather the other way round . . . that this is reinforced and so on, so it is connected, it absolutely is. (Participant 10)

The participants described that their jaw and neck pain could be connected to pain elsewhere in the body such as a painful hip joint. They mentioned that the pain had often started at a certain location and then spread such as upwards from the neck to the orofacial region or downwards from the face to the neck region.

### Seeking Affirmation and Legitimacy from Health Care

The participants expressed a desire to be seen and understood by health care providers. They described that living with pain might not be visible to health care professionals as well as not objectified by investigations such as X-rays.

The doctor did this X-ray and found no damage but the pain is there. It is not visible on the X-ray. (Participant 2)

This invisibility resulted in a need to present yourself as deserving and sensible when seeking care. The participants explained that if you have a visible and measurable reduced function, for example, if you cannot walk, you have a greater chance of receiving medical help; whereas if you have pain, you do not receive help or understanding to the same extent.

The participants described a feeling of not being taken seriously or being believed by health care, and this could result in a feeling that they were implicitly accused of being liars. The participants expressed that once they sought help, their experiences were negative with the initial points of contact (predominantly in primary care). The health care professionals did not focus on what the patients asked for, and they felt they were not properly examined or listened to regarding their thoughts and perspectives. This delegitimization as a patient occurred with health care professionals in both primary care and dentistry. However, when they were referred to a specialist within secondary care, rather than health care centers within primary care, their experience changed to be more positive. Once participants were in contact with a specialist, they felt they were often properly examined and followed-up more frequently. Some perceived that they had been validated by the specialists, in addition to the specialist seeing their situation and confirming that the pain was real.

The participants expressed a need for explanation and mentioned that they started questioning if their pain was in fact real when they did not receive confirmation or explanation from their initial contacts with health care. They believed that knowing the reason or cause of the pain would be helpful, and not receiving a diagnosis or an explanation of the cause resulted in not receiving any treatment. Participants mentioned that health care sometimes rules out different diagnoses but that they as patients did not fit in anywhere. In addition, participants also questioned whether health care professionals actually knew how to help them.

I don’t know if it’s ignorance or . . . if they take it seriously. Or maybe it’s a combination of ignorance and around pain that you don’t see? (Participant 6)

When seeking care, participants wanted to be asked about their thoughts and expectations and wanted someone to take time and listen. Furthermore, they wanted to discuss options with their health care provider. In addition to wanting a diagnosis, participants wanted to know about other patients with the same symptoms. The reason for this was to obtain information about what has helped other patients with the same symptoms and thereby to share knowledge.

The participants mentioned strategies for getting help when seeking care. They expressed that other patients with more “serious complaints” were prioritized and that they sometimes waited a few days longer to seek help with a hope of being taken seriously at that point. They also mentioned that they were aware of staff shortages and lack of time in both health care and dentistry. Moreover, the participants expressed the need to be flexible as a patient, for example, to be able to come on a short notice or be very active and contact the clinics frequently. The lack of time in health care and the fact that their pain management was not perceived as a priority might result in a need to exaggerate symptoms as a patient to get help.

The primary care service or the medical care center . . . it is almost like you need to exaggerate your symptoms to get the help you need. (Participant 1)

In addition, another strategy mentioned was the importance of being clear and prepared when explaining symptoms and concerns to health care. Some also expressed that they did not dare seek help again due to previous unpleasant experiences.

### Wishing for Clear and Easily Navigable Health Care Pathways

To choose where to seek help was something that the participants experienced as difficult. The choice between health care or dentistry was dependent on why they were in pain, and therefore, seeking care was based on where the pain or symptoms were localized. Participants perceived that dentists mainly know teeth and that jaw pain was more difficult for dentists to manage. They believed that the orofacial region belongs to dentistry, whereas the rest of the body belongs to health care. Some other examples described during the interviews were that if you have a headache you seek medical care, and if you have neck pain you see a physiotherapist. Some participants expressed that they thought their jaw pain initially was toothache and therefore sought help within dentistry. The participants mentioned that they often started seeking care at health centers in primary care.

With the jaw, it was the dentist. I thought it could be due to a wisdom tooth or infection or cavity, so that’s why I chose the dentist. (Participant 9)

The participants expressed that their dentist and medical doctor did not communicate, and there was an overall lack of communication and collaboration between different medical specialists. Some examples were that the dentist recommended seeking help from a medical doctor; but once participants sought help from the medical doctor, the opposite occurred, with the medical doctor suggesting to seek care from a dentist. This resulted in patients becoming messengers between different specialists or health care providers.

It’s like I am the one who has to keep everyone informed, like, now neurology has said this . . . and then I go there and tell them what neurology said, and there is a risk that I misunderstand or miss important information that they might need, like, that they need share with each other. (Participant 15)

The participants expressed that health care and dentistry were not aware of the other’s competence and knowledge, and they believed that a main reason for the lack of collaboration between health care and dentistry was due to time and money as well as being part of different organizations. A wish for a better collaboration between medical specialties was expressed to simplify health care navigation. The participants had been seeing several different health care providers throughout the years and wished that health care had a holistic approach regarding their situation.

## Discussion

During the interviews, participants expressed thoughts regarding their own sensemaking in terms of the causes of and reasons for their jaw and neck pain. In addition, participants had a holistic approach regarding their pain and general health that also included their mental health. Unfortunately, this holistic approach was not reflected in health care once they sought help, and care seeking did not result in receiving a confirmation or explanation of the cause. Moreover, navigating within the health care system with concurrent jaw and neck pain was perceived as challenging, mainly due to not knowing where to seek help and to the lack of collaboration between different health care providers.

In terms of understanding, participants expressed an interest in understanding the reason for their pain and wanted an explanation for the cause of their jaw and neck pain. Before seeking care, the patient’s own sensemaking regarding their pain had often begun. Approximately 60% of patients seeking primary health care in general do so because of pain (Wiitavaara et al. 2017), and the reasons for deciding to seek care varies between individuals. Pain is also the most common reason for emergency dental visits (John et al. 2020). TMD patients express that they seek care once their situation becomes unbearable in terms of symptoms and consequences, unfortunately, and in line with our findings, the patients’ expectations are not met once they seek care (Ilgunas et al. 2023).

Participants wanted to find the cause because they felt this information would help them get help and become better in the future. Moreover, participants expressed an interest in knowing about what had helped other patients. This finding is similar to results from a study from the United Kingdom, in which patients with chronic pain expressed an interest in helping other patients and for other patients to not have to go through the same experiences as they had (Hartley et al. 2023). This highlights the importance of peer support via patient groups in pain management since navigating health care with chronic pain can be both difficult and challenging.

Our results showed that participants often have their own explanation for the cause of their pain. Even if asked about perspectives on pain development, the participants often returned to the start of the pain and possible causes. This is in line with previous research, for example, adolescents with TMD pain express that they try to understand their TMD pain (Nilsson and Willman 2016), and this was also seen among patients with persistent joint-related TMD pain, where the individuals expressed a need for wanting answers (Dinsdale et al. 2022). The understanding of pain among pain patients is important, and educational interventions for explaining pain exist (Moseley and Butler 2015). Moreover, educational interventions can decrease catastrophizing and reduce pain (Moseley and Butler 2015). The reason for a patient wanting to understand their pain might be part of their own sensemaking but also a result of not receiving help or legitimization from health care. This highlights the need for health care professionals to focus more on information and educational interventions in pain management.

The participants expressed that confirmation and receiving a diagnosis were important, both of which have value in terms of the patient’s own understanding and receiving treatment. What many patients have in common when seeking care, regardless of the reason for seeking care, is that they are looking for confirmation from their health care provider. Among chronic TMD patients, receiving a diagnosis and being believed are the most important factors according to a recent meta-synthesis (Taimeh et al. 2023). Similarly, in a qualitative study following patients from an acute whiplash injury to a state of chronic whiplash, validation was perceived as important in addition to matching expectations between health care and the patients (Ritchie et al. 2017). Unfortunately, many pain patients do not receive confirmation in terms of a diagnosis from health care. According to a qualitative study in TMD patients, not receiving a diagnosis was mentioned as resulting in uncertainty (Durham et al. 2010), and this was also expressed by patients with lower back pain (Costa et al. 2023). This is in line with our results, in which participants expressed that they started questioning whether their pain was real and that they struggled with navigating within health care and not knowing where to seek help next. Another qualitative study of persistent orofacial pain showed that the patients continued to move between different health care providers but still did not receive a diagnosis and that this affected their lives negatively (Breckons et al. 2017). In line with our findings, patients with neck and back pain express that they want confirmation in terms of being taken seriously by their health care provider (Stenberg et al. 2012). The interaction between the health care provider and the patient is important for the patient and their care (Costa et al. 2023). The results of a reflexive thematic analysis study resulted in the theme “medical merry-go-around,” illustrating that patients with TMD describe that they are not receiving explanations regarding their pain (Penlington et al. 2024). As a consequence, the patients start questioning their own role in their pain situation.

Our participants believed that their jaw and neck pain was connected. Interestingly, they also believed that there was an interconnectedness between their mind and body. Pain affects both mental and physical health (Turk et al. 2016). This is supported by the fact that a brief psychological assessment is recommended at the first visit for TMD patients (Visscher et al. 2018). The participants believed that their pain was connected to their mental health and vice versa, but a mismatch seemed to exist between the patient and the health care provider in terms of pain management. In addition, pain onset was often related to stressful life events, which is in line with previous findings in which stressful events were risk factors for pain spreading (Tanguay-Sabourin et al. 2023); this underlines the importance of a holistic perspective in management. Having a holistic approach and assessing psychological factors among pain patients is also of importance in pain management since negative affectivity and pain interference among patients can affect perceived validation/invalidation from their physicians (Edlund et al. 2017).

More than 30 y ago, a diagnostic instrument for TMD, the Research Diagnostic Criteria for Temporomandibular Disorders (RDC/TMD), was launched. Despite several updates in terms of diagnostic criteria and guidelines for management of TMD during the past decades, psychosocial factors are often not considered, and the diagnostic tools are not used (Sharma et al. 2020). Therefore, the consensus among experts in the field of orofacial pain is implementation of new guidelines at their universities, education to students and colleagues, and reaching out to stakeholders.

Our participants expressed satisfaction once they were seen by a specialist. The satisfaction could be due to specialists having more knowledge regarding pain management or that they took time to listen and validate the patient. In a Swedish qualitative study, general dental practitioners expressed an interest in helping TMD patients, but in the decision-making, both structural challenges within dentistry as well as the dentist’s own competency regarding management were limiting factors (Ilgunas et al. 2021). Uncertainty regarding diagnosis also exists among dentists in primary care in the United Kingdom, which often results in referrals to secondary care (Durham et al. 2007). An increased collaboration between primary care and secondary care could be helpful as well as an increased collaboration between different medical specialties. An increased interdisciplinary collaboration between dental care and health care is also dependent on politics and stakeholders acknowledging TMD pain as a health concern. Moreover, implementation of joint methods for triage in health care and dental care could facilitate such collaboration.

Seeking care seems to become more difficult if you have pain at multiple sites due to the need to consult with more than 1 health care provider. In a recent qualitative study, patients with neck pain expressed that they felt trapped in a multifactorial problem that was difficult to get out of and that this needed to be addressed (Kragting et al. 2024). According to our knowledge, the present study is the first qualitative study of concurrent jaw and neck pain. Interestingly, our patients had a holistic approach to their pain and how it ideally should be managed. The participants expressed that the collaboration between dental and medical care is almost nonexistent and that they wished for simplified health care navigation. Furthermore, the symptom location seems to guide the patient, which further highlights the need for increased collaboration between medical and dental care to help guide the patient. Living with chronic pain can result in fear of movements, avoidance, and catastrophizing (i.e., a vicious circle) (Westman et al., 2011). Confirmation and early interventions are important to break this vicious circle that can lead to chronification so as to reduce the risk of fear of movements and catastrophizing.

Some patients are more likely to seek care, and most research is performed among care-seeking patients. In a qualitative study comparing care seekers with TMD with non–care seekers, the care seekers were critical of the available care and believed that health care held the answers, whereas the non–care seekers had low confidence in the available care and preferred to talk, for example, to their friends (Rollman et al. 2013). Our study can be considered to have been performed among care seekers, and satisfaction was perceived to be larger among participants once they met specialists. The reason for this was that once they met a specialist, participants were more often legitimized, which illustrates the importance of confirmation and having a holistic approach as a health care provider in pain management.

### Strengths and Limitations

A strength of our study is the robust interview data collected through individual interviews with a substantial variation in gender, age, education level, and pain duration among the participants in order to seek a depth and breadth of their experiences (Dahlgren et al. 2019). Individual interviews are suitable for sensitive data regarding individual health, in which the patients are free to express their thoughts and opinions. All interviews were performed in a setting preferred by the participants. If the interviews were performed at the hospital, the interviews were performed in a conference room and not in a clinical setting. All interviews were performed by a single interviewer (A.B.) who was not involved in the care of the participants. The interviewer used a pretested interview guide (Dahlgren et al. 2019) and checked all transcribed data for accuracy in addition to adding quotes and descriptions.

The researchers had different expertise, backgrounds, and nationalities. As with research in general, a researcher’s background can have implications on the interpretation of the results. In this regard, we used triangulation (Lincoln and Guba 1985) between the researchers to minimize potential bias due to different backgrounds per se. In addition, the researchers were involved in different steps and at different levels throughout the analyses. Such provision of an insider perspective due to specific expertise and knowledge of TMD and concurrent jaw and neck pain, and an outsider perspective with expertise on the methods and on pain rehabilitation have been suggested as a valid approach to reduce bias in qualitative research. Credibility was further supported due to prolonged engagement (Lincoln et al. 1985), with some members of the researcher group working at a specialist clinic in dentistry and thus having a deep understanding of the patient group and the research area. However, these researchers were not involved in the participants’ care.

The interviews were analyzed using QCA, a systematic and flexible method suitable to analyze qualitative data including multifaceted and sensitive phenomena (Graneheim and Lundman 2004). The interviews were analyzed in the Swedish language in the first stage and later translated to English by a professional translator. Our findings have been discussed and negotiated among all authors before reaching a final result, which helps guard against overinterpretation of the results and improves trustworthiness. Thus, trustworthiness was ensured through accuracy in the research design, data collection, and analyses.

We believe that our findings are transferable for specialist dentistry clinics in Sweden and other comparable countries. Although Swedish health care is considered to be of a high standard globally, management of TMD in Sweden is still far from optimal. Accessing health care in countries with less developed systems can be assumed to pose even greater challenges for patients. Since recruitment was performed at a specialist dental clinic for orofacial pain, the patients had been referred either from medical care or dental care; thus, the patients could be regarded as care-seeking patients. The participants might have had more complaints or symptoms, and therefore, the results would have differed if non–care-seeking patients would have been interviewed.

## Conclusion

Our results suggest that a mismatch exists between the patient’s own sensemaking regarding the cause of their jaw and neck pain and the care they receive from their health care provider. Having pain from multiple sites that is managed in different medical systems makes it difficult to navigate within health care. This difficulty highlights the importance of a holistic approach and a clear care pathway to reduce the time the patients spend navigating the health care system. A clear care pathway could reduce the risk of pain chronicity, and an increased collaboration between dentistry and medical care would be desirable. In addition, our findings underline the importance of provision of information both for validation and the patients’ understanding.

## Author Contributions

A. Böthun, contributed to conception and design, data acquisition, analysis, and interpretation, drafted and critically revised the manuscript; A. Fjellman-Wiklund, F. Hellström, A. Lövgren, contributed to conception and design, data analysis and interpretation, critically revised the manuscript; J. Durham, B. Häggman-Henrikson, contributed to data interpretation, critically revised the manuscript. All authors have given final approval and agree to be accountable for all aspects of the work.
